# Synthesis and Evaluation of Herbal Chitosan from Ganoderma Lucidum Spore Powder for Biomedical Applications

**DOI:** 10.1038/s41598-018-33088-5

**Published:** 2018-10-02

**Authors:** Li-Fang Zhu, Zhi-Cheng Yao, Zeeshan Ahmad, Jing-Song Li, Ming-Wei Chang

**Affiliations:** 1 Key Laboratory for Biomedical Engineering of Education Ministry of China, Zhejiang University, Hangzhou, 310027 PR China; 2 Zhejiang Provincial Key Laboratory of Cardio-Cerebral Vascular Detection Technology and Medicinal Effectiveness Appraisal, Zhejiang University, Hangzhou, 310027 PR China; 3 Leicester School of Pharmacy, De Montfort University, The Gateway, Leicester, LE1 9BH UK

## Abstract

Chitosan is an extremely valuable biopolymer and is usually obtained as a byproduct from the shells of crustaceans. In the current work, chitosan is obtained from an herbal source (*Ganoderma lucidum* spore powder (GLSP)) for the first time. To show this, both standard (thermochemical deacetylation, (TCD)) and emerging (ultrasound-assisted deacetylation (USAD)) methods of chitosan preparation were used. The obtained chitosan was characterized by elemental analysis, XRD (X-ray diffraction), FT-IR (Fourier transform infrared spectroscopy) and thermogravimetric measurements. The process resulted in chitosan possessing comparable values of DD, [η] and $\,\overline{{\rm{Mv}}}$ to the commercial product. Chitosan obtained *via* both processes (TCD and USAD) displayed excellent biocompatibility; although the USAD prepared biopolymer exhibited significantly improved fibroblast (L929 cell) viability and enhanced antibacterial zones for both *Escherichia coli* (*E. coli*) and *Staphylococcus aureus* (*S. aureus*). The findings of new herbal chitosan mark key developments of natural biomaterials; marking a potential shift from conventional sea-based organisms.

## Introduction

Chitin is a white linear polysaccharide and is insoluble in most common solvents^1^. It is the main constituent of exoskeleton in several insects and shellfish (*e.g*. crabs and shrimps)^2^. It’s well documented biodegradability and low toxicity have led to extensive use of the refined form in biomaterial fields^3^. Based on the molecular (chain) structure of *N*-acetyl-D-glucosamine linked by *β*- (1–4) glycosidic bonds, chitin is classified into three different polymeric forms; *α*, *β* and *γ*^4^. Since most abundantly used chitins are from seafood shells, which are more likely to contain allergenic contaminants and greater mineral content compared to fungi^5^; complex purification methods are often deployed to minimize consumer health risks^6,7^. Furthermore, obtaining high quality chitin from discarded and spoiling seafood shells is also challenging with further impact on production time and health risk aspects.

Chitosan is a derivative of chitin; obtained after deacetylation. In this instance, the GlcNAc unit in chitin is converted to GlcN forming chitosan^8^ with the repeating units of both biopolymers. Like chitin, chitosan is a biopolymer which has generated extensive interest; surpassing research interest in cellulose^4^, largely due to its better aqueous solubility^9^. Chitosan possesses good biocompatibility, biodegradability and low toxicity^10^ and the materials absorptivity of heavy metal ions also makes it a valuable biopolymer^11^. For these reasons it has been used in numerous fields including food^12^, pharmaceutics and biomedical engineering^13^.

Due to increased demand for chitosan (emerging bio-applications and preference over existing materials) acetylation methods and processing of chitin have diversified^14^. Conventionally, thermochemical deacetylation (TCD) is used deploying high concentrations of aqueous sodium hydroxide^15^, elevated process temperatures (120 °C) and lengthy reaction times (~4 h)^16^. To overcome this, several methods converting chitin to chitosan (with greater product yield and quality) have been developed which include microwave assisted deacetylation^17^, ultrasound assisted deacetylation (USAD)^16^ and supercritical fluid extraction^18^. USAD has proven to be a crucial breakthrough, converting *β*-chitin into chitosan (with 83–94% deacetylation) at reduced synthesis temperatures (50–80 °C) and quicker reaction times (10–60 min), which also minimizes severe depolymerization risk^19^. Chitosan is used in numerous industrial applications and is conventionally sourced from sea organisms^20^. In this regard, alternative sourcing of chitosan together with enhanced processing will provide timely developments.

**Figure 1:**
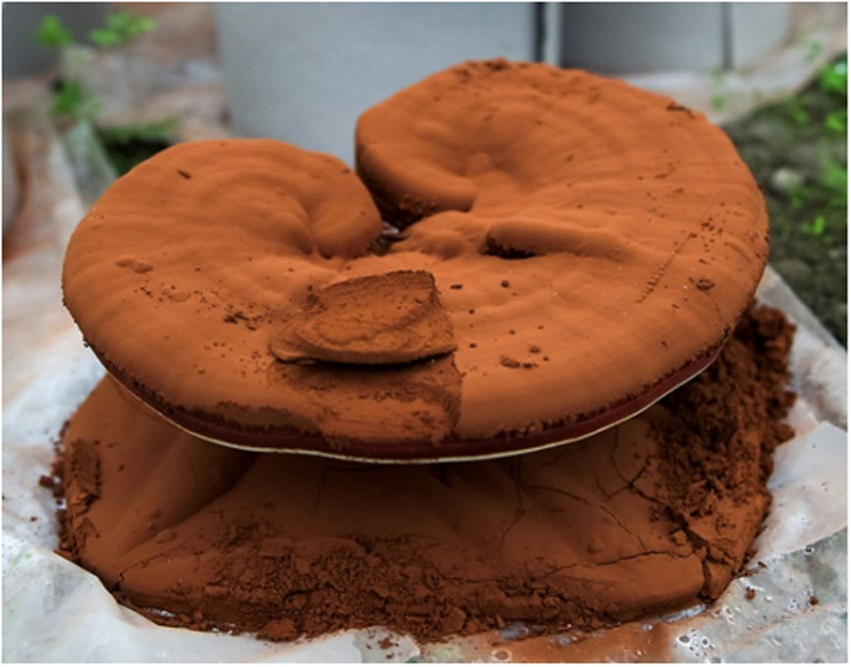
Digital image of *Ganoderma lucid* powder.

*Ganoderma lucidum*, also known as the “Linghzi” Mushroom, belongs to a large group of fungi called polypore and has been used as a medicinal product for many millennia^21^. Furthermore, potential health benefits of *Ganoderma lucidum* spore powder (GLSP, Fig. 1) have recently been documented which include improved immuno-regulation, anti-inflammatory action, anti-cancer properties, radical scavenging ability and prevention of diabetes^22^. The powder constitutes a chitin bilayer structure with an overall particulate size range of ~6–12 μm. The GLSP component comprises polysaccharides, triterpenoids, nucleotides, sterols, steroids, ganoderic acids and proteins^22^, making this a potential nutritional source for various bioactive elements. To date several raw chitin products have been developed using mainly seafood byproduct sources (crab, shrimp and lobster shells) although with little to no emphasis on unlocking the nutritional resource following human consumption. Taking into account the growing demand (and applications) of the material, alongside its nutritional composition, it becomes imperative to explore opportunities to make such bioactive components accessible.

In this study, chitin is derived from the herbal source (GLSP) for the first time. Chitosan synthesis is demonstrated using both TCD and USAD processes. A comparison between process conversion efficiency is shown and a detailed parametric study on USAD preparation from GLSP is detailed. The impact of USAD and TCD on herbal sourced chitosan is reflected through comparison with commercially obtained chitosan. The extracted chitosan was characterized by employing FT-IR, TGA, XRD, and SEM. The bioassay indicates an exciting development for sourcing an important biopolymer which is crucial and timely for several biomass and bio-based industries.

## Results and Discussion

### Deacetylation of Thermochemical Deacetylation (TCD) and Ultrasound-Assisted Deacetylation (USAD)

GLSP is an excellent source of chitin, indicating potential utilization and benefits in the biomedical and therapeutic arena. However, its conversion to chitosan from the raw herbal powder remains to be explored. In this study, conventional TCD and emerging USAD processes were utilized to obtain chitosan from fungal spores.

**Figure 2:**
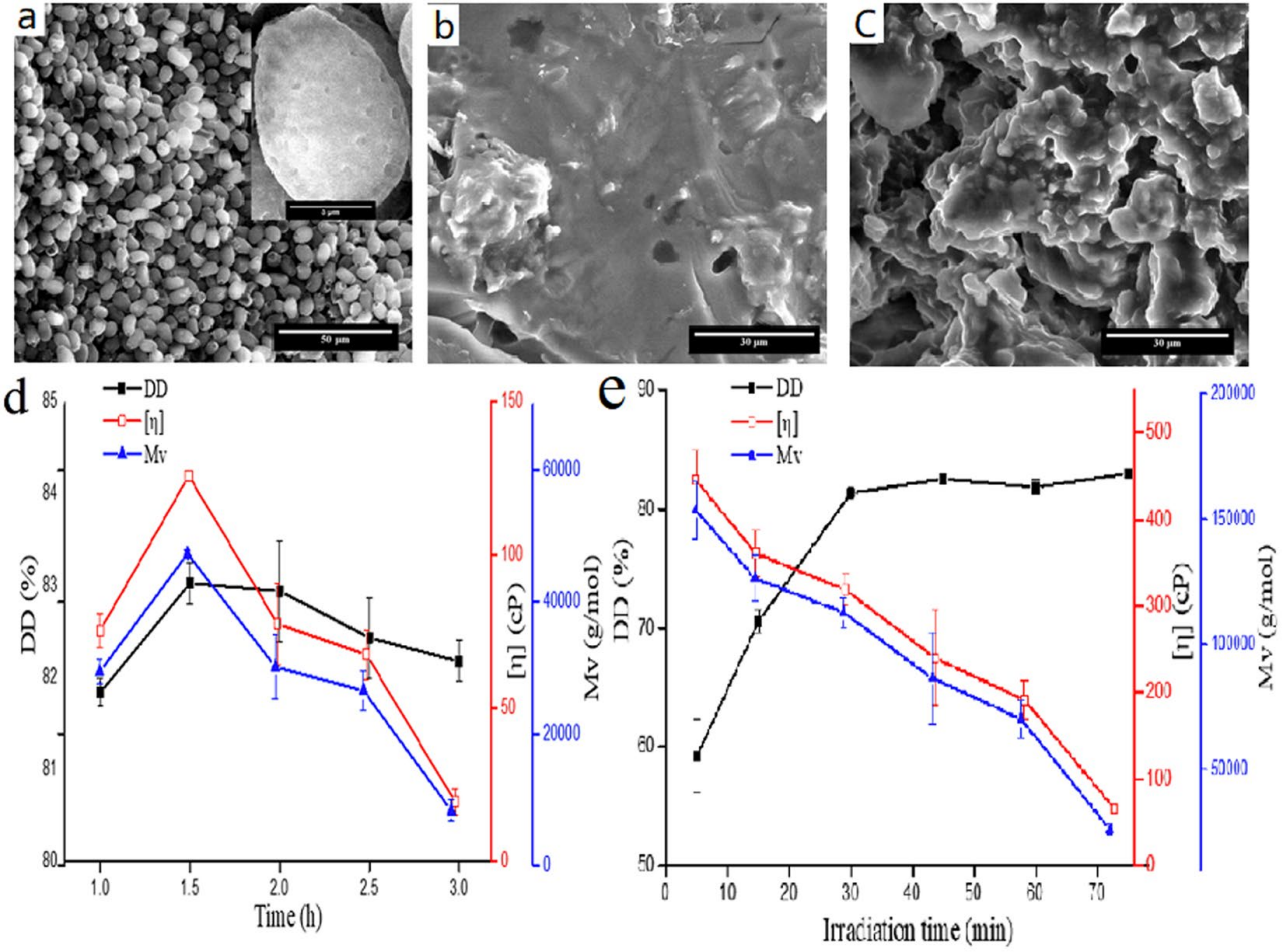
The effect of deacetylation and SEM images of GLSP, C-T and C-U (**a**) GLSP SEM (**b**) SEM of C-T (**c**) SEM of C-U, and the effect of deacetylation time on DD, [η] and $\overline{{\rm{Mv}}}$ using TCD (**d**) and USAD (**e**). C-U sample was generated using USCD setup as: 90 W, 15 min, 20 w/v% NaOH, 1:15 (g: mL).

The chitosan morphological were changed following deacetylation using both methods (Fig. 2). Although many minute pores are observed on GLSP surface, the overall particulate structure is oval in shape and rigid (Fig. 2a), which suggests the polysaccharide extraction process does not impact chitin structure. However, morphological differences arose upon deacetylation and varied depending on the method deployed. The chitosan morphology post TCD and USAD (Fig. 2b,c), were lost the well-defined oval shape. The surface chitosan obtained using TCD (termed as C-T hereafter) is relatively smooth when compared to chitosan obtained using USAD (termed as C-U hereafter). C-T was obtained using solvent and heating based reaction under static conditions, whilst C-U involved vigorous ultrasound cavitation. In this regard, the USAD method broadens application scope of chitosan obtained from the herbal source since the irregular surface is beneficial; increasing the contact area with other materials.

The effect of deacetylation time on DD, [η] and $\overline{{\rm{Mv}}}$ after TCD of GLSP is shown in Fig. 2d,e. However, for ultrasound radiation, the DD value increased significantly from 59.2% at 5 min to 82.9% at 75 min (Fig. 2e). These findings indicate the USAD method provides more linearity and control over DD, [η] and $\overline{{\rm{Mv}}}$ values for the conversion process. The magnitude of DD, [η] and $\overline{{\rm{Mv}}}$ for chitosan obtained *via* TCD is relative to chitosan obtained using UASD; although the former method of preparation requires extended reaction times and temperatures. Therefore, the USAD process is more efficient compared to the TCD method.

### FTIR and XRD analysis

**Figure 3:**
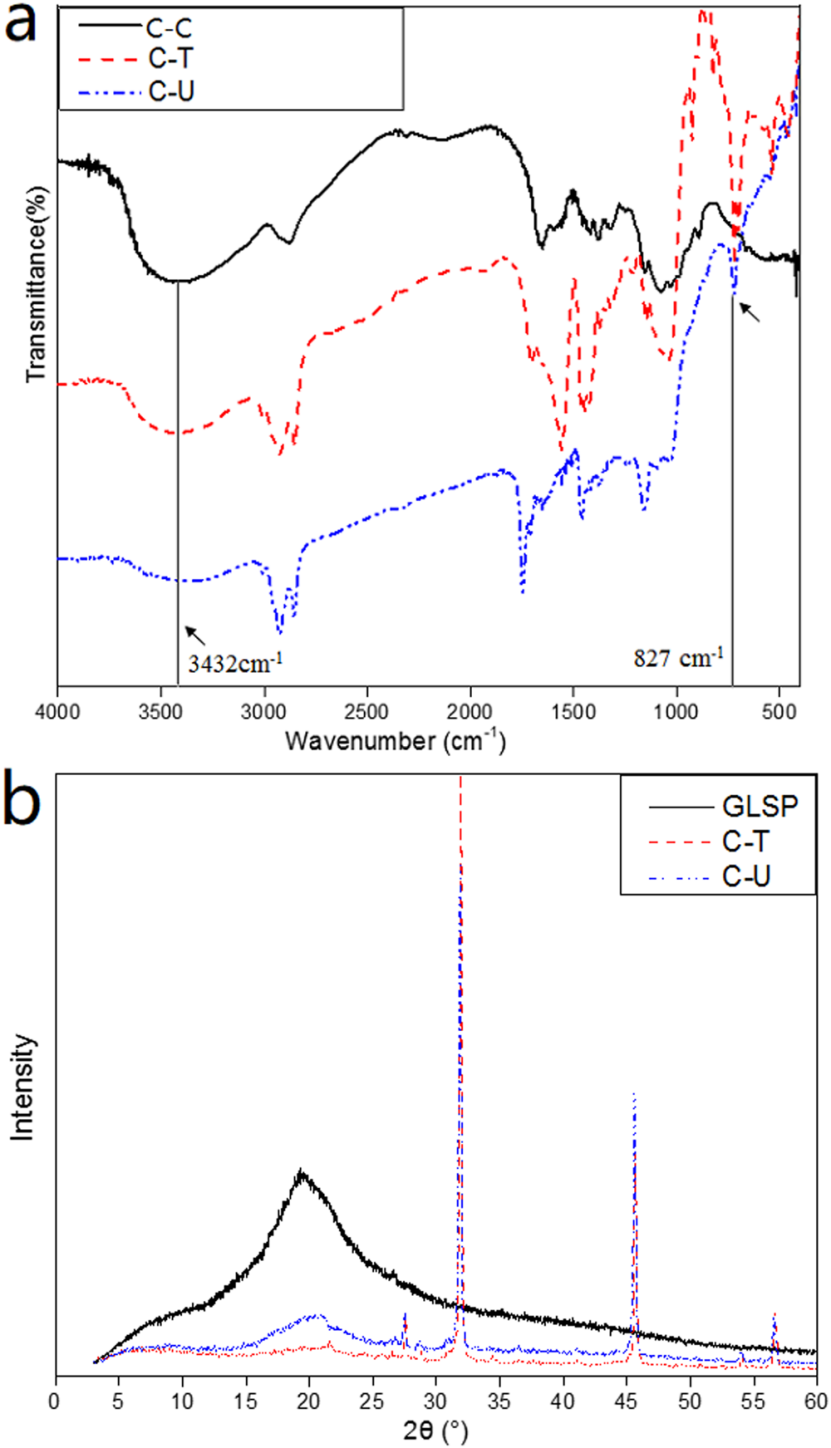
FTIR spectra of C-C, C-T and C-U (**a**); XRD analysis of GLSP, C-T and C-U (**b**).

As results showed in Fig. 3a, both TCD and USAD processes permit GlcNAc to GlcN unit conversion; converting chitin to chitosan. Characteristic peaks of C-C (commercial chitosan) were compared to C-T and C-U. The comparatively lower conversion efficiency of TCD, compared to USAD, is evident as non-characteristic peaks are detected at 450 cm^−1^ and 750 cm^−1 ^^23^. In addition, the band arising at 872 cm^−1^ is attributed to the transmittance peak of β-(1,4) glycosidic bond of chitosan, and vibrations of O-H and N-H are typically found in the 3432 cm^−1^ region^24^. The amide I band is regarded as a key characteristic band for chitosan, and for α-chitosan this occurs at 1656 cm^−1^ and 1621 cm^−1^, while for β-chitosan this is found at 1626 cm^−1^ ^25^. The amide II band for *α*- and *β*-chitosan is found at 1556 cm^−1^ and 1560 cm^−1^, respectively^26^. Hydrogen bonding between N-H and C=O group occurs in the 1656 cm^−1^ region^25^. The band split at 1621 cm^−1^ implies hydrogen bonding involving the C=O is linked to a hydroxymethyl group due to the residue from adjacent chitin.

As shown in Fig. 3b, GLSP diffractogram shows an intense peak at 2θ = 20.2°, which is regarded as the plane (020, 110)^27^. When compared to XRD data for C-T and C-U, peak intensities at 2θ = 20.2° appear much lower suggesting a reduction in crystallinity following deacetylation. Furthermore, between processed GLSP groups, peak intensity (2θ = 20.2°) for C-T is lower compared to C-U, indicating TCD reduces crystallinity to a greater degree than USAD. Spectra of both chitosan materials show four clear peaks which are observed at 2θ = 27.5, 31.86, 45.64 and 56.64° for C-T and 2θ = 27.5, 31.86, 45.58 and 56.54° for C-U. The conversion process causes peaks to shift toward higher 2θ values^28^ and new intense peaks arise upon formation of chitosan^29^, indicating a change in polymeric chain structure. According to DD values (Table 1), the maximum peak intensity at 2θ for GLSP is much higher compared to C-T and C-U which is consistent with previous work^30^. The CrI (crystalline index) (Table 1) of chitosan was lower than that of GLSP, which means the reduction of crystallinity triggered by deacetylation^31^.

**Table 1:** The DD, CrI and 2θ of GLSP, C-T and C-U.

Sample	Results		
	DD (%)	CrI (%)	2θ (°)
GLSP	25.1	56.8	20.2,
C-T	85.1	24.6	20.2, 27.5, 31.9, 45.7, 56.6
C-U	82.2	27.5	20.2, 27.5, 31.9, 45.6, 56.5

### Thermogravimetry analysis (TGA)

**Figure 4:**
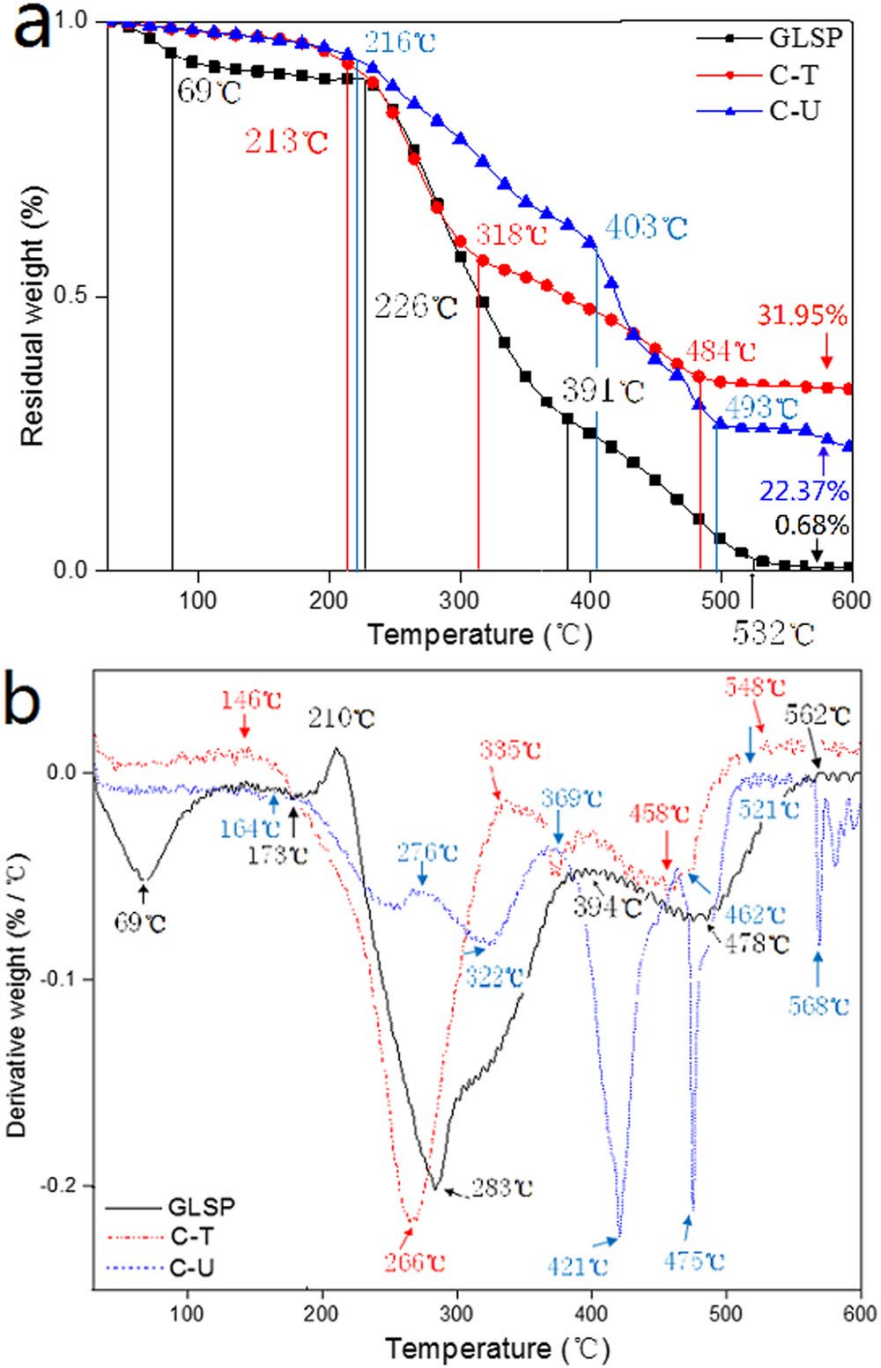
TGA analysis for GLSP, C-T and C-U. (**a**) Change in residual weight *vs*. temperature, (**b**) derivative weight *vs*. temperature.

TGA results for GLSP, C-T and C-U are shown in Fig. 4. A three stages weight loss is noted for all materials, which is in accordance with previous findings^32^. There was no distinct difference in thermostability between the three samples, with the outset degradation temperature (the temperature at which sample weight starts to decrease) of GLSP, C-T and C-U being 193, 193 and 210 °C. For the GLSP sample, ~11.45% weight loss occurred at the first stage (between 30 and 226 °C), which is ascribed to surface absorbed water and water bound to polymeric chains^31^. When the temperature increased to 391 °C, ~62.15% weight loss for GLSP sample was noted, which is ascribed to degradation of saccharide and desacetylated chitin units^5^. ~25.72% weight loss occurs at the third stage (between 391 and 532 °C) which is due to polymer decomposition. The results for C-T and C-U show a similar trend. Although, weight loss during last two stages for both C-T and C-U samples arise from saccharide degradation^32^; distinct differences between weight of the final residue between all three samples was noted. These differences are due to chitin, and its protein and crude fat content^33^. These components are found within GLSP but are removed during the deacetylation process. Hence, the residual weight post deacetylation of C-U was greater than that of C-T.

Figure 4b shows temperature related degradation on residue content for GLSP, C-T and C-U. Five peaks allude to the degradation velocity of chitin: 69, 210, 283, 394 and 478 °C, which indicate irregular degradation velocity upon temperature increase (from 30 to 600 °C). When the temperature is increased to 562 °C, the degradation velocity remains constant signaling complete degradation of chitin. Derivative weight *vs*. temperature curves for C-T and C-U indicate degradation velocity for both samples is not constant. Complete degradation for C-T and C-U was noted at 548 and 521 °C, respectively. These results indicate the thermostability of chitosan is not altered during the deacetylation process, although degradation velocity is subject to variation.

### Biocompatibility assessment

**Figure 5:**
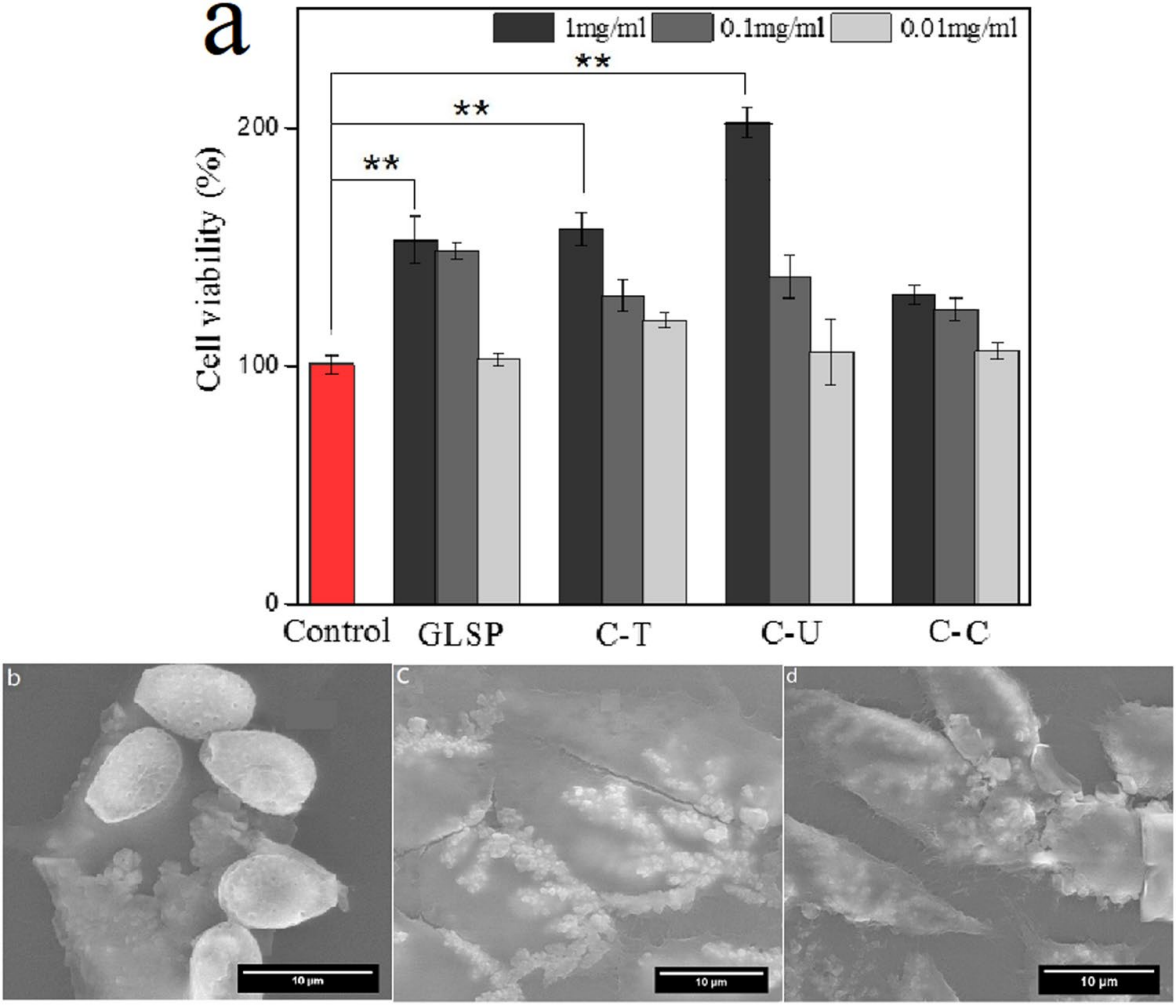
Cell viability evaluation. (**a**) Effects of GLSP/C-T/C-U/C-C with different concentration (1 mg/mL, 0.1 mg/mL, 0.01 mg/mL) on the L 929 cell viability using CCK-8 assay (**b**–**d**) SEM images of L929 cell morphology treated by GLSP/C-U/C-T (1 mg/mL). Each treatment condition was repeated using 8 wells (96-well plate).

**Figure 6:**
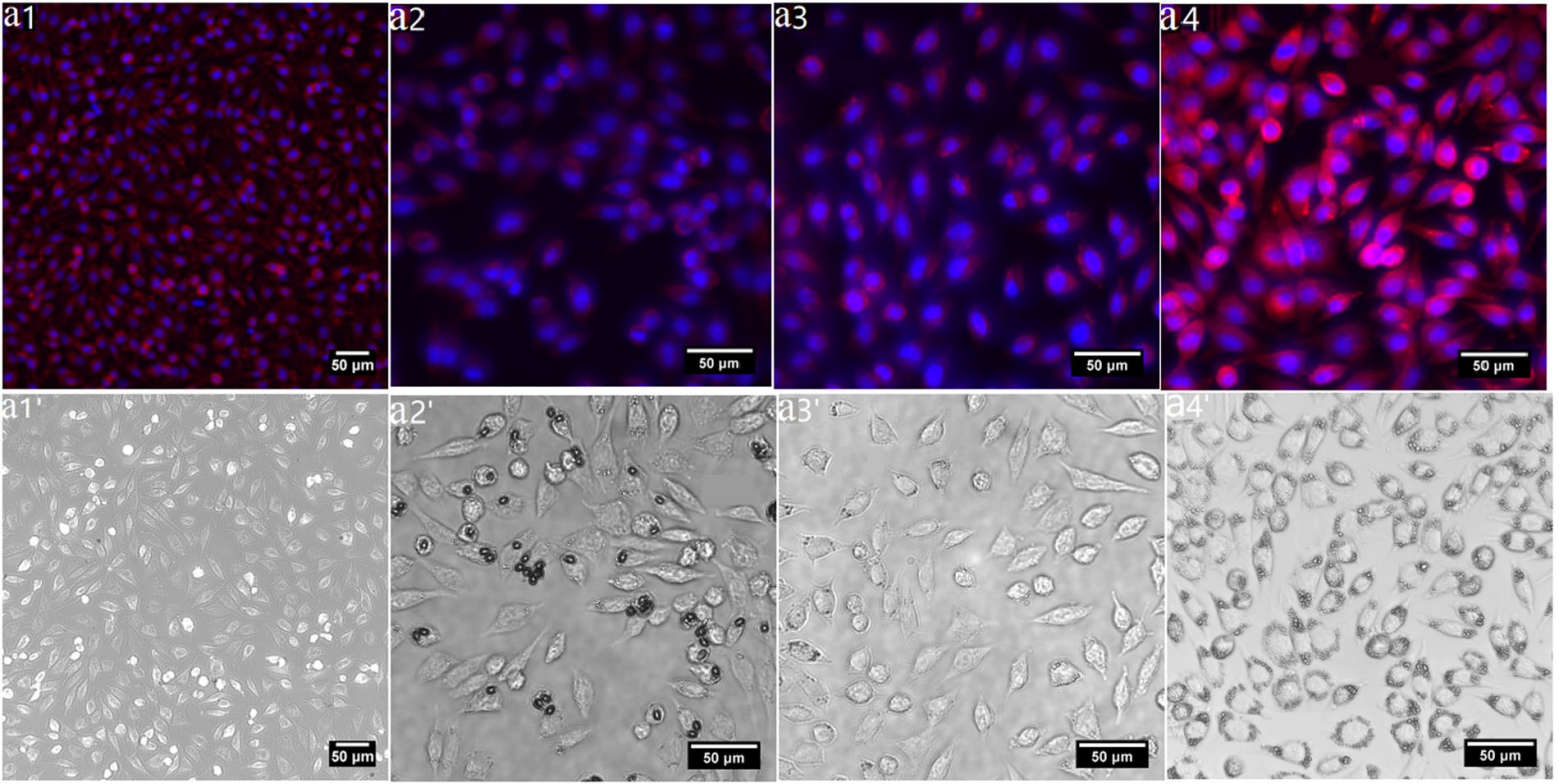
Biocompatibility assay using L929 cell. (**a1**–**a4**) Merged fluorescent images of L929 cell morphology treated by C-C/GLSP/C- U /C- T (1 mg/mL); (**a1′**–**a4′**) bright-field fluorescent image of (**b1**–**b4**) respectively.

The effect of GLSP, C-T and C-U on mouse fibroblast cell viability was assessed. Cell viability was tested using CCK-8 analysis. The addition of GLSP, C-T, C-U and C-C to MEM improved cell viability, but with varying levels compared to the control group (Fig. 5a). The addition of C-U improved cell viability rate compared to GLSP and C-T at a sample concentration of 1 mg/mL. The addition of GLSP, C-T or C-U improved cell viability significantly (T-Test for GLSP, C-T and C-U at p < 0.01). The underlying mechanism enhancing GLSP or chitosan action on L929 cell viability is due to mediation of specific α-L-rhamnose recognizing lectin-site triggered stimulation of olgo- and polysaccharides. This mechanism also includes collagen biosynthesis^34^. The SEM images (Fig. 5b–d) showed the GLPS or chitosan adherence to cell surface, which confirmed the biocompatibility of chitosan further. The C-T adhered to cell surface individually, and the shape of C-T was nubby nearly, which is most likely due to crystallization of C-T in culture medium. C-U adhered to the cell surface with bunchy but also dense form. This may be attributed to the rough surface avails the adherence of cell, since surface roughness of the C-U product was greater than the C-T sample. The reduced size and rougher surface enhanced adherence to cells surface. Although proliferative acceleration of polysaccharides from abalone on HepG2 cells has been reported previously^35^, specific impact of GLSP, C-T and C-U on cell viability needs to be investigated further, to evaluate these as better biomaterials when compared to chitosan obtained from conventional seafood byproducts. The merged and bright-field fluorescent micrographs (Fig. 6a1–a4′) showed intact cellular structures comprising cell nuclei and cytoskeleton. The results indicate that the addition of GLSP/chitosan improved cell viability, which provided excellent biocompatibility which is ideal for a potential biomass.

### Antibacterial activity

**Figure 7:**
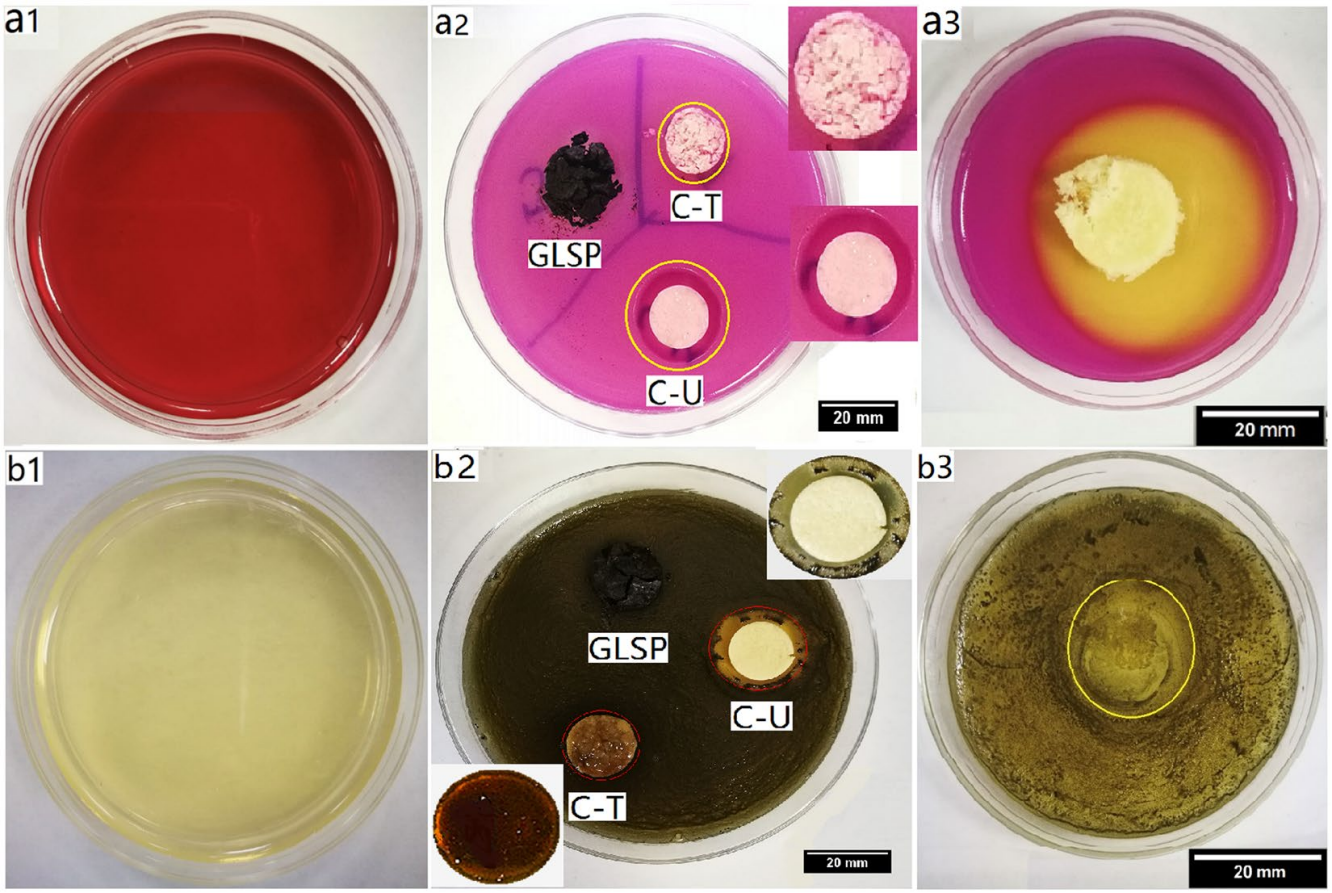
Effect of antibacterial activity assay. (**a1**) Violet red bile agar plate. (**a2**) Antibacterial effect of GLSP, C-T and C-U on *E. coli* post 24 h inoculation at 37 °C (**b1**) baird-parker agar plate. (**b2**) Antibacterial effect of GLSP, C-T and C-U on *S. aureus* post 24 h inoculation at 37 °C (**a3** and **b3**) antibacterial effect of C-C on *E. coli* and *S. aureus*, respectively.

Antibacterial properties of GLSP, C-T, C-U and C-C were evaluated using the agar diffusion method. *E. coli* (Gram −) and *S. aureus* (Gram +) were selected as model bacteria. Antibacterial properties were quantified based on the inhibition zone surrounding the circular disc samples (Fig. 7). The inhibitory effect of C-U on both *E. coli* and *S. aureus* appears to be much greater than C-T, which may be due to shorter C-U molecular chains which favor penetration into bacterial cells (Table 2).

**Table 2:** Inhibition zone obtained using agar plates method against *E. coli* and *S. aureus*.

Sample	Inhibition zone diameter (Mean ± SD, mm)		
	*E. coli*	*S. aureus*	Level
GLSP	0	0	/
C-T	16.9 ± 0.1	16.4 ± 0.2	*
C-U	23.8 ± 0.1	21.3 ± 0.1	**
C-C	43.8 ± 0.2	21.1 ± 0.3	**

*Very sensitive, **extremely sensitive.

Changes to *E. coli* and *S. aureus* cytoplasmic membrane integrity post treatment with C-T or C-U was analyzed using a fluorescence microplate reader *via* PI and FDA staining. PI dye is useful for non-viable cell detection and binds with DNA and fluoresces red. FDA passes through membranes and accumulates within cells and fluoresces green; making it ideal for cell viability. Both FDA and PI were used to provide a more accurate quantification of cell viability and analysis^36^.

**Figure 8:**
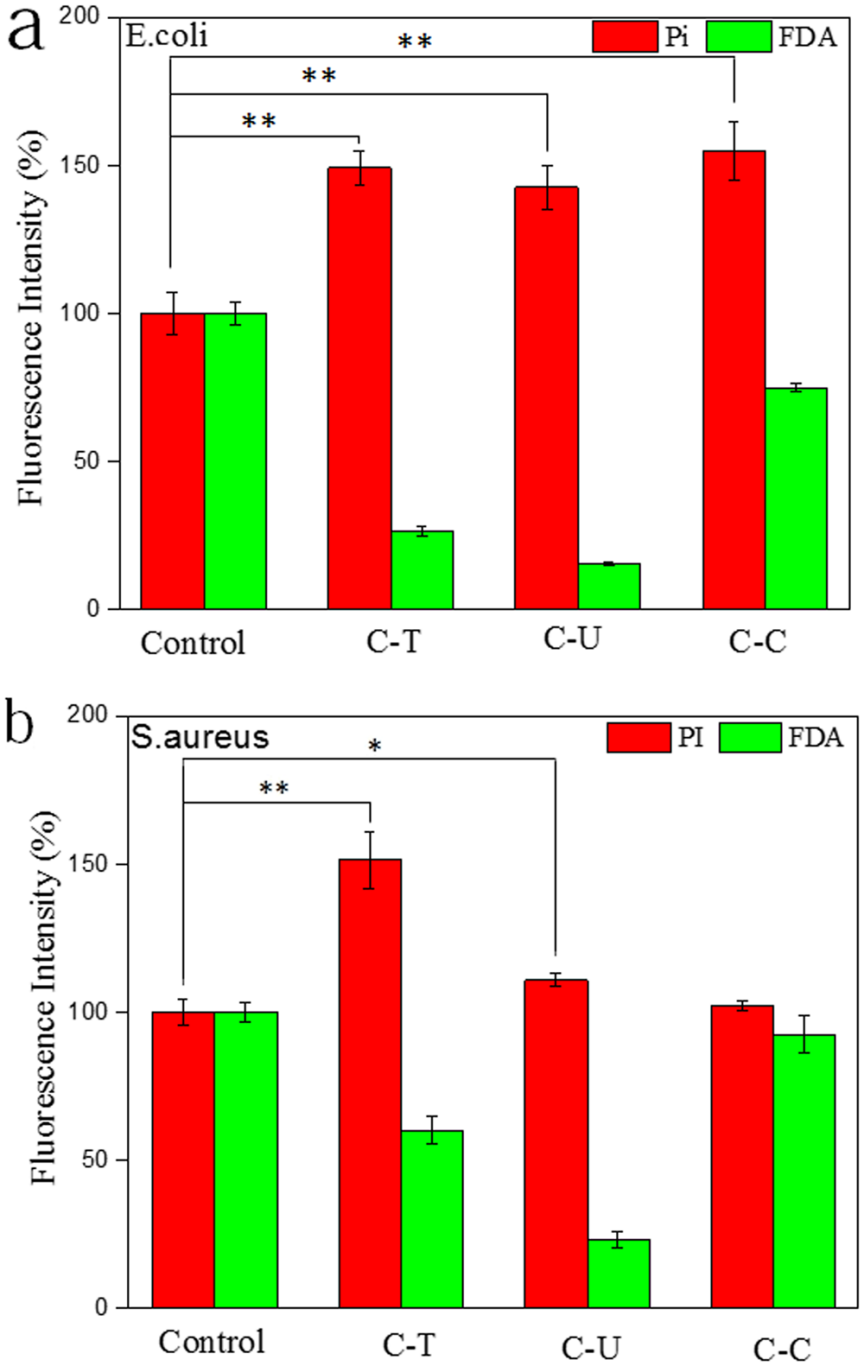
Fluorescence microplate reader analysis of membrane permeability. (**a**) Staining of *E. coli* using Pi and FDA (**b**) staining of *S. aureus* using Pi and FDA; 0.4 mL inoculum containing ~1.5 × 10^6^ CFU/mL of either *E. coli* or *S. aureus* were incubated in each well (96-well plate) at 37 °C for 24 h. Identical concentration of chitosan (1 mg/mL) was added to each well. Eight wells were measured from each test group, **p < 0.01, *p < 0.05.

Incubation of *E. coli* with C-T or C-U (at 1 mg/mL) led to a reduction in the proportion of living cells from 100% (control group) to 26.38 and 15.34% (Fig. 8a), respctively. The proportion of non-viable cells increased from 100% (control group) to 179.08 and 142.53%, respectively. The results (Fig. 8b) reveal that addition of C-T or C-U at a concentration of 1 mg/mL is suffecient to inhibit growth of *S. aureus*. Compared to the control group, reductions from 100% to 60.05 (C-T group) and 23.05% (C-U group) were achieved. The proption of non-vialble cells increased from 100% to 151.39 (C-T group) and 110.81% (C-U group).

Antibacterial and antifungal activities of chiosan have been reported^37^. Inhibitory action towards gram-negative bacteria (*e.g. E. coli*) is attributed to chitosan binding on outer cell membranes leading to an increase in bacterial cell permeability. This in turn causes leakage of fundamental biological compounds^38^. Inhibitory action towards gram-positive bacteria (*e.g*. *S. aureus*) is linked to nutrient entry restriction in to cells; leading to cell death^39^. Fluorescence intensities of PI or FDA indicate antibacterial effects of C-T were greater than C-U. This is attributed to a greater DD value obtained for C-T (as shown in Table 1); which is related to several parameters known to affect antibacterial activity such as molecular weight, DD, structure and positively charged content etc^40,41^.

## Conclusion

The current work investigates the herbal based source for chitosan production. Both TCD and USAD successfully induced N-deacetylation reaction and converted chitin into chitosan and products were compared with a commercial source. The obtained chitosan were characterized by elemental analysis, XRD, FT-IR and thermogravimetric measurements. Chitosan derived using both processes displayed excellent biocompatibility and improved fibroblast cell viability and antibacterial activity. Compared to control and TCD groups, chitosan prepared *via* USAD displayed better viability and antibacterial zones. These interesting findings indicate an exciting breakthrough for herbal based chitosan for use in biomaterial science.

## Materials and Methods

### Materials

GLSP was provided by TianHe Agricultural Group (Zhe Jiang Long Quan, China). GLSP relics were used in this study following polysaccharide extraction as shown in previous work^42^. Hydrochloric acid, acetic acid, sodium chloride (troche) and sodium hydroxide (troche) were obtained from Sinopharm Chemical Reagent Co., Ltd. (Shanghai, China). Modified eagle’s medium was obtained from (MEM, Gibco, USA) and fetal bovine serum (FBS) was obtained from Sijiqin (Sijiqin, Hangzhou, Zhejiang, China). Deionized water (DI) was produced using a Millipore Milli-Q Reference ultrapure water purifier (Millipore, Bedford, USA). Hydrogen peroxide 30% (H_2_O_2_) was obtained from Sinopharm Chemical Reagent Co., Ltd. (ShangHai, China). All chemicals were analytical grade without additional purification. For comparison, standard (commercial) chitosan (C_6n_H_11n_NO_4n_) was purchased from Macklin (Macklin Biochemical Co., Ltd., Shanghai, China).

### Preparation of GLSP sediment

GLSP relics obtained post polysaccharide extraction were dried in a vacuum oven (−0.095 MPa, D2F-6020AF, Tianjin GongXing Laboratory Instrument Co., Ltd., Tianjin, China) at 65 °C for 3 days prior to further treatment. Dried GLSP was then bleached with 30% H_2_O_2_ at 70 °C for 2 h according to a previous method^43^. The pH of resulting suspension was adjusted to neutral using NaOH and universal indicator paper (pH 1–14, Shanghai SSS reagent Co., Ltd., Shanghai, China). Subsequently, the mixture was centrifuged at 7000 rpm for 10 min (Centrifuge 5810 R, Eppendorf, Germany), after which the GLSP sediment was transferred into a centrifuge tube and stored for further experimentation.

### Thermochemical deacetylation (TCD) process efficiency

The GlcNAc unit in chitin is converted to GlcN forming chitosan with the repeating units of both biopolymers. In TCD process, NaOH solution (20 w/v%) was prepared by dissolving NaOH flakes in DI water. The alkali solution was then added to GLSP sediment (0.5 g) at a solid-liquid ratio of 1:15 (g: mL) into a conical flask. Individual GLSP suspensions were then heated at 90 °C (Thermostatic Water Bath, DK-8D, Jinghong Laboratory Instrument Co., Ltd., Shanghai, China) for pre-determined time periods (from 1 to 3 h). Subsequently, HCl was added in drop-wise fashion to adjust the pH, after which neutral suspensions were allowed to stand overnight (14 h). Suspensions were then centrifuged at 7000 rpm for 10 min and resulting sediments were collected and dried by in a vacuum oven (0.95 MPa, D2F-6020AF, GongXing Laboratory Instrument Co., Ltd., Tianjin, China) at 65 °C for 3 days, which was final product (chitosan).

### Ultrasound-assisted deacetylation (USAD) process efficiency

NaOH solution (20 w/v %) was prepared by dissolving NaOH flakes in DI water. The alkali solution was then added to GLSP sediment (0.5 g) at a solid-liquid ratio of 1:20 (g: mL) into a centrifuge tube. Several identical suspensions were prepared and subjected to ultrasound irradiation for 5 to 75 min at the ambient temperature (25 °C) using the ultrasonic transducer (Branson Digital Sonifier 250, Danbury, Conn., USA). During experimentation, the temperature of deionized water was monitored every 5 min using a mercurial thermometer (MC, 30 cm, 0 °C–200 °C). The actual temperature of irradiated sample varied with irradiation time (Supplementary Fig. S1). For ultrasound irradiation process, the device was equipped with a ultrasonic probe (diameter = 1.3 cm, ν = 20 kHz). The distance from ultrasound probe to centrifuge tube was maintained at 2 cm, while the total volume of DI water (2 L) occupying the glass utensil (20 × 15 × 15 cm) was also kept constant. Following ultrasonic irradiation, HCl acid was added in dropwise fashion to suspensions until neutrality was obtained. Suspensions were then allowed to stand overnight (14 h). Resulting samples were subjected to centrifugation and sediments were collected and dried in a vacuum oven (−0.095 MPa, D2F-6020AF, Tianjin GongXing Laboratory Instrument Co., Ltd., Tianjin, China) at 65 °C for 3 days, after which the final product (chitosan) was obtained.

### Morphology assessment

Field emission environmental scanning electron microscopy (SEM, Quanta FEG650, FEI, China) were used to study surface features of raw GLSP and resulting chitosan products. For SEM analysis, an accelerating voltage of 20 kV was used. Samples were fixed on metallic stubs using double-backed conductive tape. Prior to analysis, all samples were sputter-coated (108 Auto Cressington Sputter Coater, Ted Pella, INC.) with a thin layer of gold under vacuum for 60 s using a current intensity of 25 mA.

### DD, [η] and $\overline{{\bf{Mv}}}$ measurements

The DD (%) of chitosan was obtained using Eqs (1) and (2) as reported by Brugnerotto *et al*.^44^:

1 $${{\rm{A}}}_{1320}/{{\rm{A}}}_{1420}=0.3822+0.03133\,{\rm{DA}}$$

2 $$\mathrm{DD}( \% )=1-{\rm{DA}}$$

where DA is degree of acetylation, A_1320_ and A_1420_ absorbance values obtained at 1320 and 1420 cm^−1^, respectively.

Chitosan was added to NaCl (40 mL, 0.2 M) and acetic acid (0.1 M) at a 1:1 solution volume ratio. The suspension was then subjected to ultrasound at the ambient temperature (25 °C) for 1 h. A viscometer (DV2TLVCJ0, Brookfield, USA) was used to determine [η] at 25 ± 0.5 °C. Each sample was measured in triplicate. [η] was calculated using Eqs (3–5)^45^:

3 $${\eta }_{sp}=\frac{{\rm{\eta }}-{\eta }_{0}}{{\eta }_{0}}$$

4 $${{\rm{\eta }}}_{{\rm{r}}}=\frac{{\rm{\eta }}}{{{\rm{\eta }}}_{0}}$$

5 $$[\eta ]=\frac{1}{c}\sqrt{2({\eta }_{sp}-\,\mathrm{ln}\,{\eta }_{r})}$$

where,*η*_0_ is solvent viscosity, η is solution viscosity, *η*_*sp*_ is a specific viscosity value and *η*_*r*_ is the ratio of *η*_*r*_ and *η*_0_.

$\overline{{\rm{Mv}}}$ (viscosity average molecular weight) was calculated from [η] using the Mark-Houwink equation, shown as Eq. (6)^46^, where κ, α are variable parameters dependent on solution and temperature^47^.

6 $$[{\rm{\eta }}]={{\rm{\kappa }}\bar{{\rm{M}}}v}^{{\rm{\alpha }}}$$

here, the values κ = 1.81 × 10^−3^ L/g and α = 0.93 were used to calculate viscosity average molecular weight $(\overline{{\rm{Mv}}})$. Since $\overline{{\rm{Mv}}}$ is reduced upon chitin conversion to chitosan, polymer distribution and agglomeration are also affected. Therefore, a decrease in $\overline{{\rm{Mv}}}$ serves as a useful indicator for the chitosan conversion process.

### Fourier transform infrared spectroscopy (FT-IR)

FT-IR spectroscopy (IR Affinity 1, Shimadzu, Japan) was used to confirm presence or absence of functional groups in materials. Samples were prepared using the KBr pellet pressing method^48^, and a scanning range of 4000–400 cm^−1^ was selected. In brief, 2 mg of C-C, C-T and C-U were mixed and grinded with ~200 mg KBr powder using a pestle and mortar. Mixtures were compressed into transparent pellets using powder compressing machine (FW-4A, TUOPU instrument Co., Ltd., Tianjin, China) under pressure (14 MPa) for 2 min. Spectra for each sample was acquired from 20 scans at a resolution of 4 cm^−1^.

### Thermogravimetric analysis

TGA was performed under atmospheric conditions using a TGA/DSC1 device (Mettler-Toledo, UK). A heating step rate of 10 °C/min was selected, and the temperature ranged from 31 to 600 °C. TGA was performed on C-C, C-T and C-U, with the initial weight of the three samples recorded as 2.94, 2.94 and 3.03 mg, respectively.

### X-ray diffraction (XRD)

XRD analysis was performed at the ambient temperature (25 °C) on an X-ray diffractometer (Gemini A OHra, Oxford Varian, UK). Samples were measured using 1°of DivSlit and 10 mm of DivH.L.Slit, and examined at 40 kV/30 mA. Data was obtained in the 2*θ* range 3–60° at continuous scanning deploying a step of 0.02° at a step speed of 5°/min. The CrI was calculated using the following Eq. (7) ^16^:

7 $${{\rm{CrI}}}_{110}=[({{\rm{I}}}_{110}-{{\rm{I}}}_{{\rm{am}}})/{{\rm{I}}}_{110}]\times 100$$

where, I_110_ and I_am_ are the maximum diffraction and amorphous diffraction intensities at 2*θ* ≈ 20° and 13°, respectively.

### Cell culture and viability assay

Cell viability was assessed using CCK-8 (cell counting Kit-8 reagent, Dojindo Laboratories, Kumamoto, Japan). L929 cells were cultured in modified eagle’s medium (MEM) (Gibco, Carlsbad, California US) supplemented with 10% fetal bovine serum (FBS, Gibco) in 6 cm diameter cell culture dishes, under standard conditions at 37 °C in a humid atmosphere (5% CO_2_) for 48 h. A cell suspension (density = 1.4 × 10^5^cells/mL) was obtained. Selected materials were disinfected using UV irradiation for 2 h before addition to cell culture medium. 100 μL of cell suspension was pipetted into a 96-well plate, which was incubated at standard conditions for 24 h. GLSP, C-T, C-U or C-C (1, 0.1 and 0.01 mg/mL of each) were added to a 96-well plate and incubated at standard conditions for 24 h. 10 μL CCK-8 solution was then added and the absorbance value from 8 wells (post 4 h incubation, for each condition) was assessed using a microplate reader (spectra Max 190, NanoDrop, USA) at 450 nm. In addition, 1 mL of the original cell suspension was added to 2 mL MEM directly into a cell culture dish (Φ = 3 cm) and incubated at standard conditions for 24 h. The incubating medium was then removed and 3 mL of fresh medium was added along with 3 mg of GLSP, C-T, C-U or C-C and incubated for 24 h. Fluorescence microscopy (Olympus, BX61W1-FV1000, Tokyo, Japan) was used to observe the impact of biopolymer growth on cells. Alexar Fluor 546 phalloidin (Invitrogen, Carlsbad, California USA) and 4′,6′-diamidino-2-phenylindole hydrochloride (DAPI, Invitrogen) staining reagents were used. Cell treatment was performed according to a previous method^49^ prior to fluorescence microscopy. Stained cells were analyzed further using SEM to determine cell and biopolymer interaction. For SEM analysis, samples were coated with a layer of gold as described in 2.3.1, although an accelerating voltage of 15 kV was used.

Control groups consisted of cells incubated with MEM and CCK-8. Blank groups comprised mixtures of MEM and CCK-8 only. Cell viability in the control groups were defined as 100% and cell viability in treated groups was calculated using the following Eq. (8):

8 $${\rm{Cell}}\,{\rm{viability}}\,( \% \,{\rm{of}}\,{\rm{untreated}}\,{\rm{cells}})=[({\rm{As}}-{\rm{Ab}})/({\rm{Ac}}-{\rm{Ab}})]\times \mathrm{100} \% .$$

Here, As, Ac and Ab are the absorbance values for experimental, control and blank groups, respectively.

### Antibacterial activity

Antibacterial activity of GLSP, C-T, C-U or C-C was based on a plate assay method. Inhibition zone assay was used to indicate antibacterial properties against two common pathogens; *E. coli* ((NW1014 (8099), Nanjing Maojie Microbiology Technology Co. Ltd., Jiangsu, China) and *S. aureus* (CMCC (B)26003, Shanghai Luwei Microbial SCI. & TECH. Co., Ltd., China). 300 mg of selected biopolymers (*e.g*. GLSP, C-T or C-U) were pressed into discs using a compressing machine (FW-4A, Tianjin TUOPU instrument Co., Ltd., Tianjin, China). 0.4 mL inoculums containing ~1.5 × 10^6^ CFU/mL of either *E. coli* or *S. aureus* were added to violet red bile agar (Qingdao Hope Bio-Technology Co., Ltd., Shandong, China) plates and baird-parker agar base (Qingdao Hope Bio-Technology Co., Ltd., Shandong, China) plates, respectively. Egg-yolk tellurite emulsion was then added to baird-parker agar base culture system (Qingdao Hope Bio-Technology Co., Ltd., Shandong, China). Compressed GLSP, C-T or C-U discs were placed on inoculated agar plates (10 cm diameter) after which they were incubated (SHP-080 Biochemical Incubator, Shanghai Jinghong Laboratory Instrument Co., Ltd., Shanghai, China) at 37 °C for 24 h. The diameter of inhibition zone was measured for all samples.

### Fluorescence microplate reader analysis

0.4 mL inoculum containing ~1.5 × 10^6^ CFU/mL of either *E. coli* or *S. aureus* were incubated in nutrient broth (Qingdao Hope Bio-Technology Co., Ltd., Shandong, China) and 7.5% sodium chloride broth (Qingdao Hope Bio-Technology Co., Ltd., Shandong, China), respectively. *E. coli* and *S. aureus* were stained using 25 μg/mL propidium iodide (PI) (Solarbio Life Science, Beijing, China) for 20 min and 25 μg/mL fluorescein diacetate (FDA) (Solarbio Life Science, Beijing, China) for 20 min, respectively, at the ambient temperature (25 °C) prior to fluorescence analysis. Fluorescence analysis was carried out using Fluorescent microplate reader (FlexStation II, NanoDrop, USA). Control groups comprised bacteria incubated in broth only for 24 h at 37 °C.

### Statistical analysis

All experiments were performed in triplicate and data is given as mean ± standard deviation (n = 3). Statistical analysis was performed using SPSS software (SPSS Statistics v18, IBM, UK). All statistical data was plotted using Origin software (OrginLab, USA).

## Electronic supplementary material

Supplementary Information
