# Supplementary material for: Synthesis and Evaluation of Herbal Chitosan from Ganoderma Lucidum Spore Powder for Biomedical Applications
Source: Sci Rep. 2018 Oct 2;8:14608. doi: 10.1038/s41598-018-33088-5 (PMC6168458; doi:10.1038/s41598-018-33088-5)
Supplement: Supplementary file 1 — Supplementary Information [file 41598_2018_33088_MOESM1_ESM.docx]

Synthesis and Evaluation of Herbal Chitosan from Ganoderma Lucidum Spore Powder for Biomedical Applications

Li-Fang Zhu ^a,b^, Zhi-Cheng Yao ^a,b^, Zeeshan Ahmad ^c^, Jing-Song Li ^a^, Ming-Wei Chang ^a,b,*^

^a^ Key Laboratory for Biomedical Engineering of Education Ministry of China, Zhejiang University, Hangzhou, 310027, PR China.

^b^ Zhejiang Provincial Key Laboratory of Cardio-Cerebral Vascular Detection Technology and Medicinal Effectiveness Appraisal, Zhejiang University, Hangzhou, 310027, PR China.

^c^ Leicester School of Pharmacy, De Montfort University, The Gateway, Leicester, LE1 9BH, UK.

*corresponding author: Ming-Wei Chang, Ph.D., Assoc. Professor

Tel: +86(0)571-87951517, Email: mwchang@zju.edu.cn


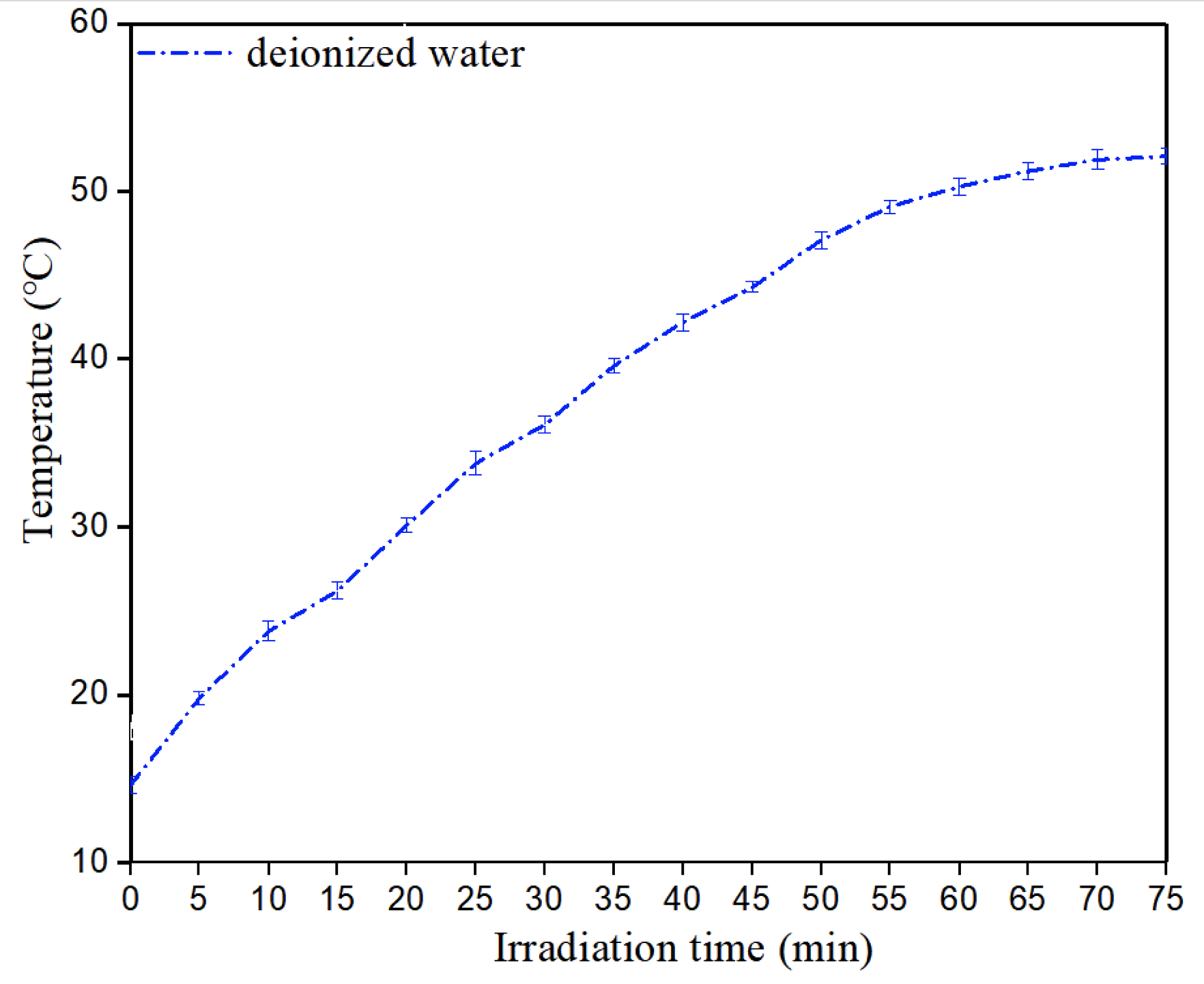


Supplementary Figure S1: temperature change to water tank during ultrasound irradiation.
